# Chemical Toxicants in Water: A *GeoHealth* Perspective in the Context of Climate Change

**DOI:** 10.1029/2022GH000675

**Published:** 2022-08-01

**Authors:** Naveen Joseph, Tate Libunao, Elizabeth Herrmann, Shannon Bartelt‐Hunt, Catherine R. Propper, Jesse Bell, Alan S. Kolok

**Affiliations:** ^1^ Idaho Water Resources Research Institute University of Idaho Moscow ID USA; ^2^ Department of Civil Engineering University of Nebraska Lincoln NE USA; ^3^ Department of Biological Sciences Northern Arizona University Flagstaff AZ USA; ^4^ Department of Environmental, Agricultural and Occupational Health College of Public Health University of Nebraska Medical Center Omaha NE USA

**Keywords:** *GeoHealth*, environmental justice, climate change, community‐engaged research, public health

## Abstract

The editorial focuses on four major themes contextualized in a virtual *GeoHealth* workshop that occurred from June 14 to 16, 2021. Topics in that workshop included drinking water and chronic chemical exposure, environmental injustice, public health and drinking water policy, and the fate, transport, and human impact of aqueous contaminants in the context of climate change. The intent of the workshop was to further define the field of *GeoHealth*. This workshop emphasized on chemical toxicants that drive human health. The major calls for action emerged from the workshop include enhancing community engagement, advocating for equity and justice, and training the next generation.

## Key Points

- Integrating additive and synergistic biological actions of water contaminants can address the issue of chemical exposure and health outcomes
- Environmental injustice can be mitigated by inviting community stakeholders to participate in scientific‐based policy decision‐making
- Community‐engaged research and action can facilitate policy decisions to safeguard drinking water and public health

## 1 Introduction

In recent years, there has been an upswell in research that relates earth and environmental sciences to human, agricultural, and environmental health (McEntee, 2016). The field of *GeoHealth* has attracted the attention of many water resource professionals that desire to use large, pre‐existing datasets to characterize the water quality and quantity of “waterscapes” (i.e., watersheds, streams, aquifers) as they pertain to ecosystem services and human health (Jordan & Benson, 2015). A three‐day *GeoHealth* workshop titled “Chemical Toxicants in Water: A *GeoHealth* Perspective in the Context of Climate Change” was conducted focusing on climate change, environmental health, and science communication in the context of water quality. The workshop was organized by the Center for Health Equity Research at Northern Arizona University, in conjunction with faculty from the University of Idaho, the University of Nebraska Medical Center, and the University of Nebraska–Lincoln, from June 14th to June 16th, 2021. The four major themes of the *GeoHealth* workshop were: drinking water and chronic chemical exposure, environmental injustice, public health and drinking water policy, and the fate, transport and human impact of aqueous contaminants in the context of climate change. The workshop demonstrated the importance of integrating geospatial data with epidemiological research, addressing the long‐standing issue surrounding environmental justice, and including approaches to serve communities most impacted by environmental contamination and climate change. This editorial reflects the research intentions that came out of the workshop.

## 2 Drinking Water and Chronic Chemical Exposure

Clean drinking water quality is of the utmost importance to public health, as almost 10% of the global population does not have access to safe drinking water (World Health Organization, 2015). While contaminated drinking water can result from the presence of microbes, chemical contamination can also lead to several adverse health outcomes, including increased cancer incidence, cardio‐vascular diseases, and adverse reproductive outcomes (Levallois & Villanueva, 2019; Villanueva et al., 2014; Ward et al., 2018). For instance, Ward et al. (2018) did a meta‐analysis of epidemiological studies and found linkages between an increased exposure to nitrates through drinking water and methemoglobinemia, adverse reproductive outcomes, cancer, and thyroid disease in women. Similarly, Jones et al. (2020) demonstrated the negative environmental impacts and human health risks associated with mining activities on unregulated groundwater sources in the Western Navajo Nation. While these case studies were conducted across different regions, all of them identified that populations exposed to high concentrations of chronic chemical contaminants (e.g., arsenic, uranium etc.) may also expect to see an increase in some specific adverse health impacts.

While many countries monitor drinking water quality, there is a need for a virtually continuous updating of methodologies and safe water quality standards, as advances in water quality quantification and emerging public health concerns lead to the identification of previously unregulated contaminants (Levallois & Villanueva, 2019). Furthermore, while focusing on an individual contaminant and its public health impact is beneficial (Almberg et al., 2018; Richard et al., 2014), there is also a need to shift toward focusing on synergistic interactions between contaminant mixtures (Joseph et al., 2022; Lagunas‐Rangel et al., 2022; Villanueva & Levallois, 2015).

## 3 Environmental Injustice

Environmental injustice (Cook et al., 2021; Mathiarasan & Hüls, 2021) is a long‐standing reality in which disadvantaged communities often bear a disproportional burden with respect to environmental contamination and subsequent adverse health impacts (Diaz, 2016). For example, several studies have identified that families living in socio‐economic deprived areas are disproportionately exposed to environmental threats resulting in higher susceptibility to adverse health outcomes (Bashir, 2002; Evans, 2004; Rosen & Imus, 2007). However, on a community level, their collective voices are typically not heard, and they are rarely incorporated into decision‐making processes (Diaz, 2016).

While environmental injustice undoubtedly occurs, it can be more complicated than it appears at first blush (Butler et al., 2016; Edwards et al., 2009; Wines & Schwartz, 2016). Occasionally, community members may not be receptive to information about environmental contamination and management within their community (e.g., Bennett and Dearden (2014); Gehrig et al. (2018)), particularly when authorities or residents prioritize economic impacts over environmental ones (Al‐Emadi et al., 2022; Prayag et al., 2021). This can be attributed to not wanting to lose access to convenience, property value depreciation, the fear of an increased tax burden to support new or improved infrastructure, and the tendency of communities to favor short‐term gains over longer‐term issues. One way to dampen community resistance is to engage the community in as many science and science communication steps as possible (Gray, 2018). This strategy builds trust and provides citizens with the appropriate information to make decisions that fit within their community framework (Altman et al., 2008; Nyström et al., 2018). Examples where this has been effective, identified that the approach of two‐way communication between citizens and scientists fosters collaborative work, relationship building, and learning (Mercer & Littleton, 2007; Riesch & Potter, 2014; Roche et al., 2020).

While the *GeoHealth* field promotes integrated science, there is a need to improve efforts to produce open science to ensure environmental justice (Barnard et al., 2022). Adopting open science strategies such as ICON (Integrated, Coordinated Open Network) throughout the research lifecycle helps alleviate this environmental injustice problem to an extent by empowering populations that are disproportionately marginalized (Acharya et al., 2022; Arora et al., 2022; Barnard et al., 2022; Dwivedi et al., 2022; Salman et al., 2022; Sharma et al., 2022). ICON science principles aim to (a) integrate science across physical, chemical, biological, and social attributes; (b) coordinate the use of consistent protocols across systems and researchers; (c) provide for an open exchange of ideas, data, and models throughout the research lifecycle, and (d) create a platform for multiple stakeholders to network (Barnard et al., 2022; Goldman et al., 2022).

While cohesion between scientists and the community is laudable, its impact on environmental health can be eroded if the driving force underlying the cohesion is the short‐term acquisition of funding (e.g., Fanini et al. (2019)). Community members are, by definition, in it for the long term, not for the length of a 3–5 years Federal grant (Gralton et al., 2004). Consequently, these partnerships need to extend beyond the life of a single project and focus on building sustained relationships (Lindenmayer et al., 2012). Funding schemes must be constructed to provide long‐term support for ecological issues. Likewise, integrating community stakeholders into all facets of the program can provide opportunities for sustainability and the continuation of the project beyond the initial funding mechanism (Holmes, 2011; Lindenmayer et al., 2012).

## 4 Public Health and Drinking Water Policy

Underrepresented communities, particularly those with low‐income status, are at a disproportionate risk of consuming poor drinking water (American Public Health Association, 2001). For instance, drinking water violations were more prevalent in Black and Hispanic mid‐sized communities in the United States when poverty levels exceeded 40% (McDonald & Jones, 2018; Switzer & Teodoro, 2017). Furthermore, low‐income communities have a greater tendency to have their drinking water delivered through aging infrastructure. In the U.S. alone, the American Society of Civil Engineers reported that aging water systems pose a substantial risk to public health from water piping deterioration, leading to leaching contaminant loads into potable water sources (Grigg, 2015). As an example, the *GeoHealth* workshop highlighted the significance of Flint, Michigan's historic, lead‐based water delivery systems and fixtures as primary sources responsible for the lead poisoning phenomenon.

Considering drinking water policy and public health in a global context, there are current inadequacies of formal and informal institutions to ensure safe drinking water and sanitation. For example, World Health Organization (2014) found that less than one‐third of 94 countries have policies, plans, and coverage targets set for educational institutions and health care facilities to meet and monitor safe drinking water standards. This pattern is pervasive in developing communities and correlated to a lack of funding, access to tools for monitoring, and a general infrastructure deficiency (Li & Wu, 2019; Montgomery & Elimelech, 2007).

A holistic approach to policy governance, involving government officials, professionals and the public, needs to be implemented to safeguard public health and ensure safe drinking water. These policies should include water supply and distribution regulations and measures to guarantee safe drinking water quality (Li & Wu, 2019). Examples where this has been effective include studies such as Jabed et al. (2020) and Dey et al. (2019), who reported people's perception of poor water quality and health in the coastal areas of Bangladesh and identified that most of the households were willing to pay for safe drinking water. As evidenced by these studies, large datasets involving multiple stakeholders are essential for understanding the mechanisms of drinking water quality and public health outcomes (Li et al., 2017).

Acquiring the requisite large databases can be augmented through the use of citizen scientists. Furthermore, citizen science can help to play a significant role in these studies for helping with data accumulation and analysis (Farnham et al., 2017; McKinley et al., 2017). For example, citizen science can contribute to filling water quality data gaps by collecting information in remote or geographically challenging areas, and providing the necessary data to inform or influence regulatory decisions (Hadj‐Hammou et al., 2017). For citizen science to achieve its greatest utility, regulatory agencies must develop quality assurance standards that allow crowdsourced data to be vetted and considered during policy development. Further, community‐generated data can stimulate research driven questions by providing long‐term monitoring information that would otherwise not be available to advisory committees or policy‐developing entities. By providing correct tools in the hand of citizens, communities can make independent health conscientious decisions rather than unknowingly consume contaminated drinking water. In doing so, we see progress in bridging the gap between public health and environmental health from the ground up.

## 5 Fate, Transport, and Human Impact of Aqueous Contaminants in the Context of Climate Change

Human‐induced alteration in climate is significantly associated with deteriorating physical and mental health (Meierrieks, 2021; Mullins & White, 2019). The climate features which affect health directly include extreme heat events (Lorenz et al., 2019; Oldenborgh et al., 2018), air pollution (Kinney, 2018; Orru et al., 2017; Silva et al., 2017), and extreme weather conditions (Ebi et al., 2006; Stott, 2016). However, the indirect impacts, though less obvious, also cause complex health outcomes, among which are: the spread of disease through insects, ticks, and rodents (Mosello et al., 2020; Paz et al., 2007; Short et al., 2017), contaminated water affecting food security (Gregory et al., 2005; Lake et al., 2012; Ruttan et al., 1994; Wlokas, 2008), and disruption of overall well‐being through loss of housing and/or access to clean food and water resources (Cianconi et al., 2020; Hrabok et al., 2020; Ingle & Mikulewicz, 2020; Palinkas & Wong, 2020).

Human‐induced climate change also impacts water quality, as climate change leads to water depletion and scarcity, increasing geogenic contaminant concentrations in global drinking water sources (Coyte et al., 2019; Leveque et al., 2021). Notably, climate change attributed to rising temperature, increased precipitation intensity, and prolonged droughts are found to alter the environmental fate and behavior of chemical toxicants resulting in water quality deterioration (Kundzewicz et al., 2007; Murdoch et al., 2000; Noyes et al., 2009; Zwolsman & Van Bokhoven, 2007). As an example, for the 2012–2013 field season, Lombard et al. (2021) identified that drought in the conterminous United States increased the number of individuals exposed to elevated arsenic from 2.7 million to 4.1 million people (Lombard et al., 2021). Supporting this, Aribam et al. (2021) found a strong correlation between climate change and groundwater arsenic seasonal variation.

Since the climate‐related stressors mentioned above are diverse, a multidisciplinary approach to identifying causation factors is required, as is research focusing on the identification of climate metrics that predict adverse human health outcomes (Kinney, 2018). There is also a need to prepare public health agencies through education, monitoring, and research. While some policymakers are beginning to accept climate change and its implications on human health, current policy approaches are still underdeveloped. One way to move forward is to begin integrating mitigation policies for climate change (e.g., reducing greenhouse gases) and adaptation policies for climate change (e.g., preparing the community for climate impacts), as evidenced by recent studies (e.g., Ganesh and Smith (2018)).

Supporting this, recent studies have pointed out four major principles for addressing health implications of climate change, which include mainstreaming, linked approach, population perspective, and coordination (Ganesh & Smith, 2018; Gould & Rudolph, 2015; Marinucci et al., 2014; Parker‐Flynn, 2014). Mainstreaming corresponds to integrating climate change into all planning and policy processes throughout various sectors, while a linked approach emphasizes adaptation and mitigation policies as a part of climate change. A population perspective examines the health status of populations rather than the aggregation of individuals, while overall coordination can bring together public health law and policy to mediate the health impacts of climate change (Ganesh et al., 2020; Ganesh & Smith, 2018).

## 6 Future Recommendations

Researchers need to shift toward an integrated *GeoHealth* approach with sustained inclusion of and communication with the community. As the environmental problems being faced are complex, and research teams need to tackle these issues from a multidisciplinary approach, it is critical that communication is part of the process. Since these problems do not occur in isolation but in communities of varying socio‐economic status and across broad geographies, active work is required to build *GeoHealth* capacity to understand challenges at the earth‐health interface to ensure equity (Barnard et al., 2022; Hayhow et al., 2021). It is also the mission of *GeoHealth* professionals to be allies and advocates for environmental justice by collaborating with individuals to safeguard their communities from exposure to contaminants, particularly in the era of climate change. This could be achieved by adopting open science throughout the *GeoHealth* research using tools such as ICON (Fortner et al., 2022; Goldman et al., 2022).

As academic professionals, our moral obligation is to provide the necessary training and collaborative funding for the next generation of scientists to address global issues through a transdisciplinary lens. Additionally, to address the challenge of releasing data that the public needs, we need data tools and experts who can help the public use these tools. For instance, issues like the Flint water crisis need to be addressed from an environmental and public health perspective. However, ongoing data collection efforts are not designed in a way that maximizes their utility relative to environmental preservation or the safeguarding of community public health. A poignant example of this is the field of wastewater‐based epidemiology, which has been successfully used to inform health care professionals about the spread of infectious agents, such as SARS‐CoV‐2 (Mao et al., 2020). These data are critical to protecting community health, yet the data are either not publicly released, or if they are, the implications are not made clear. Consequently, there is both a need and an opportunity to train not only the transdisciplinary scientists but also the community organizations using a convergent approach, such as *GeoHealth*, to address water‐related challenges in a manner that will benefit the environment while also addressing critical information needs in public health.

Although targeting a specific portion of the population or the “majority” can influence policy, it can also have negative consequences at the local level that lead to environmental injustices. From the *GeoHealth* perspective, it is the duty of engineers and scientists to inform communities about threats to their public health while still protecting their individual values. Together, we can change existing academic practices by integrating the *GeoHealth* perspective into the curriculum, fostering holistic multidisciplinary research, and including community‐engaged participatory research approaches. In summary, to protect our environmental resources as our climate changes, we need community‐engaged research and action along with training and funding so that the next generation can address the holistic issues that were the focus of this editorial and last year's workshop.

### 6.1 Summary of Calls for Action

The major calls for action put forward by the workshop corresponding to each of the sections are listed as follows:

1. Drinking water and chronic chemical exposure
  - Help propagate water quality methods, including methods for emerging or understudied contaminants, into places where water quality monitoring is scarce.
  - Make sure that water quality analyses continue to focus on cumulative impacts across a suite of water quality parameters.
2. Environmental injustice
  - Adopt open science strategies throughout the *GeoHealth* research using tools such as ICON science.
  - Partnerships between community members and scientists should focus on a long‐term sustainable relationship to mitigate environmental injustices.
3. Public health and drinking water policy
  - A holistic approach to policy governance, including different stakeholders, should be implemented to ensure safe drinking water quality.
  - Citizen science can be used to address the issue of big data requirement to understand the mechanism of drinking water quality and public health outcome, providing that agencies can develop suitable quality assurance protocols.
4. Climate change
  - There is a need to prepare public health agencies regarding climate change and its impact on public health through education, monitoring, research, and contribution to public dialog.
  - Four major principles to mitigate the potential implications of climate change on human health are mainstreaming, linked approach, population perspective, and coordination.

## Conflict of Interest

The authors declare no conflicts of interest relevant to this study.

## Data Availability

Data were not used, nor created for this research.
